# Risk assessment of transgender people: implementation of a demasculinizing–feminizing rodent model including the evaluation of thyroid homeostasis

**DOI:** 10.1186/s13062-023-00450-1

**Published:** 2024-01-02

**Authors:** Alessia Tammaro, Gabriele Lori, Andrea Martinelli, Luigia Cancemi, Roberta Tassinari, Francesca Maranghi

**Affiliations:** 1 Center for Gender-Specific Medicine, Istituto Superiore di Sanità, Rome, Italy; 2 Department of Biomedicine and Prevention, University of Rome “Tor Vergata”, Rome, Italy; 3 Experimental Animal Welfare Sector, Istituto Superiore di Sanità, Rome, Italy

**Keywords:** Hormone therapy, Testosterone, Estrogen, Cyproterone acetate, Risk assessment

## Abstract

**Background:**

Individuals whose gender identity differs from the biological sex and the social norms are defined as transgender. Sometimes transgender undergo gender affirming hormone therapy, which lasts for the entire life making essential to evaluate its potential long-term effects. Moreover, transgender can represent a susceptible sub-group of population and specific attention is needed in risk assessment, including the development of targeted animal models. Aim of the study is the implementation of a rodent demasculinizing–feminizing model through the setting of appropriate dose of hormone therapy and the selection of specific biomarkers to evaluate the sex transition. Specific attention is paid to thyroid homeostasis due to the close link with reproductive functions. Four male adult rats/group were subcutaneously exposed to three doses plus control of β-estradiol valerate plus cyproterone acetate at: 0.045 + 0.2 (low), 0.09 + 0.2 (medium) and 0.18 + 0.2 (high) mg/dose, five times/week. The doses were selected considering the most recent recommendations for transgender woman. Sperm count, histopathological analysis (testis, liver, thyroid), testosterone, estradiol, triiodothyronine and thyroid-stimulating hormone serum levels and gene expression of sex dimorphic CYP450 were evaluated.

**Results:**

The doses induced feminizing–demasculinizing effects: decreased testosterone serum levels at the corresponding cisgender, increased estradiol, impairment of male reproductive function and reversal of sex-specific CYP liver expression. However, the medium and high doses induced marked liver toxicity and the low dose is considered the best choice, also for long-term studies in risk assessment. The alterations of thyroid indicated follicular cell hypertrophy supported by increased thyroid-stimulating hormone serum levels at the higher doses.

**Conclusions:**

The implementation of animal models that mimic the effects of gender affirming hormone therapy is essential for supporting clinical studies in transgender people and filling data gap in order to ensure an appropriate risk assessment and a more accurate, personalized care for transgender people.

## Background

Gender identity is each person's sense of belonging to a gender or sex. It is an individual experience, where each person can identify her/his self as a woman, a man, both, neither, or anywhere along the gender spectrum. Gender identity can align or differ from the sex assigned at birth. Individuals whose gender identity differs from the biological sex are defined as transgender (TG), and they often undergo medical gender-affirming hormone therapy (HT) [1, 2]. The HT of TG woman consists of treatment with estrogens and antiandrogens at the same time: antiandrogens have the two important functions of lowering the testosterone (T) levels to the female range (< 50 ng/dL) and of reducing the dose of the estrogens to be used [3]. In fact, high estrogen doses suppress androgen production (via central feedback) but may be associated with strong adverse effects such as thrombosis [4]. Spironolactone in the US and cyproterone acetate (CPA) in EU are among the most popular adjunctive androgen-lowering/-inhibiting agents [5]. HT is a long-term treatment, which lasts for the entire life of the individual, so it is essential to study all possible side effects [6, 7]. In addition, like general population, TG people are exposed to environmental contaminants; [6, 8] indeed, for TG people, HT might represent an additional risk factor and the implementation of a specific animal model to address the hazard identification is of primary importance [6].

On that point, the recent in vivo study to implement a rodent model mimicking de-masculinizing feminizing (dMF) HT failed to define a reliable dose level of CPA in combination with estradiol (E2) valerate. The toxicological effects recorded during the treatment were considered not compatible with lifelong HT and they have been potentially attributable to CPA, that is known to suppress T in presence of marked hepatotoxicity [9]. In fact, the study showed that in rat 0.33–0.93 mg/dose of CPA (about 25–50 mg/dose in humans) caused hepatic steatosis, inflammation and sinusoidal dilatation [10], and all these effects are typical expression of CPA toxicity [11, 12]. Moreover, recent data provide indications that low-dose CPA treatment for TG women is as effective as high-dose treatment and possibly safer [13, 14].

In this context, the aim of the present study is the selection of the suitable dose to implement the dMF animal model based on the current data on human therapies. T serum level is measured to verify the achievement of the corresponding cisgender levels and specific functional and tissue biomarkers have been identified to characterize the model, in particular: sperm count, E2 serum levels, histopathological analysis of testis and liver, and the gene expression of sex-specific liver cytochrome P450 (CYP450) isoforms identified as biomarkers of liver demasculinization after two weeks of HT in the dMF rat model [10]. Since in TG individuals undergoing HT the endocrine system is overstimulated by the therapy, they may represent a sub-population more vulnerable and susceptible [6–8] than the general population to the effects of endocrine disrupters (EDs). EDs are defined as "an exogenous chemical, or mixture of chemicals, that can interfere with any aspect of hormone action". Several pesticides, fungicides, industrial chemicals, plasticizers, nonylphenols, metals, pharmaceutical agents and phytoestrogens are EDs and humans can be exposed by ingestion, inhalation and dermal uptake [15, 16]. Thus, EDs and HT can have mutual targets in the organism: for these reasons, the analysis of thyroid gland homeostasis in rats receiving different dosages of HT has been also included in this study. Indeed, it is known that the thyroid influences several systems such as reproductive, metabolic, nervous, etc. [17, 18], and currently no data are available on the potential impact of HT on thyroid and on the prevalence of thyroid diseases in TG population.

## Methods

### Ethical approval

The animal study was performed in accordance with the Directive 2010/63/EU, the Italian Legislative Decree n. 26 of 4 March 2014 and the Organisation for Economic Co-operation and Development Principles of Good Laboratory Practice. The study protocol was approved by the Italian Ministry of Health (authorization n° 806/2021-PR).

### Experimental design 

Sixteen young male Sprague–Dawley rats (8/9 weeks old) were purchased from Envigo (Italy). Upon arrival, animals were pair-housed under standard laboratory conditions (see [10] for further details). Pellet food and water were available ad libitum. Following two weeks of adaptation, male rats were randomly divided into four groups as follows:

- Control group (C): sesame oil (vehicle)
- Low dose (L): 0.045 mg E2 valerate + 0.2 CPA mg per dose
- Medium dose (M): 0.09 mg E2 valerate + 0.2 CPA mg per dose
- High dose (H): 0.18 mg E2 valerate + 0.2 CPA mg per dose

The drugs were administered by single subcutaneous injections (200 µL), five times a week for two weeks. The group size and the doses were calculated as reported in Tassinari et al., 2023 [10]. Briefly, the dose of E2 valerate (0.18 mg per day), after the suitable adaptation for rat, corresponds to the higher dose of 10 mg/day suggested by the main clinical guidelines for TG women [1, 19]; the dose of CPA (0.2 mg per dose) was selected considering the recent HT recommendations for TG woman, and correspond to a daily dose of 10 mg [2, 5].

During the experiment, rats were monitored twice a day (at 9:00 a.m. and 4:00 p.m.) for general health conditions. Body weight (bw) and food consumption were recorded two times a week. Twenty-four hours after the last treatment, rats were anaesthetized with a gaseous solution of isoflurane and blood samples were collected by intracardiac puncture for measuring serum hormones [T, E2, thyroid stimulating hormone (TSH) and Thyroxine (T4)]. Subsequently, animals were sacrificed by CO_2_ inhalation, and necropsy and gross pathology were performed. Liver and testes were excised and weighted to evaluate absolute and relative (organ weight/body weight*100) weight. Right epididymis was sampled and used for sperm count analysis. For histopathological analysis, liver and thyroid were fixed in 10% buffered formalin whereas, to better preserve tissue morphology, testes were fixed in Bouin’s solution. A lobe of liver was flash frozen in liquid nitrogen and stored at – 80 °C for gene expression analysis.

### Sperm count

Right caudae epididymides were excised, rinsed with Dulbecco's Modified Eagle Medium (DMEM) medium (Gibco Rodano (MI), Italy), transferred on a Petri dish containing 1 mL DMEM medium and minced with scissors. Epididymal pieces were fluxed through a Pasteur pipette to facilitate sperm extrusion. Sperm suspension was filtered through a 200-mesh nylon to remove tissue fragments and diluted up to 10 mL. Sperm were counted by a Neubauer chamber under a light microscopy (Nikon Eclipse Ts2) [10].

### Blood collection and biochemical evaluation of hormones

Shortly before the sacrifice, blood was collected by intracardiac puncture with stratification across groups to reduce the potential impact of circadian rhythm and pulsatility. Blood samples were left to coagulate at room temperature for 1 h, centrifuged for 15 min at 2000 rpm twice in a cooled bench-top centrifuge (Microlite Microfuge, Thermo Electron Corporation) and stored at -80 °C until use. Serum levels of all hormones were measured in the same analytical section by the following commercial ELISA kits of the same lot(s):

- E2 Rat kit (RTC009R—BioVendor Brno, Czech Republic), LOD 2.5 pg/ml
- T Mouse/Rat kit (RTC001R—BioVendor Brno, Czech Republic), LOD 2.5 pg/ml
- TSH Rat Kit (ELK2283—ELK Biotechnology, China), LOD 0.071 ng/mL
- T4 Rat Kit (ELK8716—ELK Biotechnology, China), LOD 1.42 ng/mL

Each kit provided a standard solution of the hormone and serial dilutions were prepared to derive a standard curve and define the range of linearity of each test. For all the analyses, the manufacturer’s instructions were followed. Each sample was assessed in duplicate and the absorbance was read at 450 nm on a VICTOR3 Multilabel reader (Perkin Elmer, USA). The unknown hormone concentrations in samples were derived using the standard curve of each hormone and the software GraphPad Prism 5.0 (GraphPad Software Inc.).

### Histological and histomorphometrical analysis

After fixation, liver, testes and thyroid were dehydrated in a graded series of alcohol baths and embedded in paraffin by the tissue processor (Shandon Excelsior ES, Thermo Scientific). The 5-μm-thick histological sections were prepared using the Microm HM 325 (Thermo Scientific) and stained with hematoxylin/eosin for the examination under a light microscopy (Nikon Microphot FX) [20]. The scoring of the lesions was semi-quantitative, using a 5-point grading scale (0 to 4), taking into consideration the severity of the changes based on the criteria explained by Shackelford et al. [21] and summarized in Table 1.

**Table 1:** Scores based on distribution of tissue lesions

Score	Tissue lesion severity (% affected)
Grade 0: No change	0
Grade 1: Minimal	< 10
Grade 2: Mild	11–20
Grade 3: Moderate	21–40
Grade 4: Marked	> 40

The quantitative histomorphometrical analysis were performed on testes and thyroid by means of an image analysis system (Nis-Elements BR) applied to an optical microscope (Nikon Microphot FX). Testis tubular diameters, the relative area of the seminiferous tubules and the lumen were measured in 20 randomly selected tubules (10 × objective) [10]. Thyroid morphometrical analysis were performed, according to Rasinger et al. [22] as follows: follicular density (ratio between number of follicles and a predetermined area, 10 × lens); indirect follicular cell height (mean ratio of follicle and colloid area in five randomly selected follicles/sample 40 × lens); the mean ratio of follicular epithelium areas and number of nuclei (in the same follicle to evaluate follicular maturation); direct follicular cell height (mean of five cell height in five randomly selected follicles/sample, 64 × lens).

### Gene expression analysis

Gene expression was performed on liver according to Tassinari et al., 2023 [10]. Briefly, total RNA content was extracted by liver samples with the Norgen kit (Norgen Biotek Corp. Thorold, Canada) according to the manufacturer’s instructions, RNA quantity assessed by Nabi Nano Spectrophotometer (MicroDigital Co. Ltd., Seoul, Republic of Korea) and RNA integrity (A260/A280 ≥ 1.8) evaluated by 1% agarose gel electrophoresis. RNA (1 µg) was reverse-transcribed to cDNA using the Tetro cDNA Synthesis Kit (Quantace, Mumbai, India) according to the manufacturer’s instructions. Specific primers for *Cyp2c11*, *Cyp3a18*, *Cyp2c12*, *Cyp2c6*, and glyceraldehyde-3-phosphate dehydrogenase (*Gapdh*), as housekeeping gene, were designed using the Primer-BLAST web application and purchased from Metabion (Metabion International AG, Germany). The Excel TaqTM Fast Q-PCR Master Mix SYBR (SMOBIO Technology Inc., Hsinchu City, Taiwan) was used to perform real-time PCR assays, running reactions on a Bioer LineGene 9600 (Bioer, Hangzhou, China). Experiments were performed in duplicate on 96-well PCR plates. Threshold cycles were calculated by the LineGen9620 software (Bioer). Data are expressed as ΔΔCt ± SD values for each target gene with control samples as calibrator and *Gapdh* [10].

### Data analysis

Data management and enter were performed using Microsoft Excel 2013 and analysed using the software JMP 10 (SAS Institute Inc., Cary, NC, USA). Graphics were designed using the GraphPad Prism 5.0 software (GraphPad Software Inc., San Diego, CA, USA). Body weight, food consumption, absolute e relative organ weight, hormone serum levels and tissue morphometrical and gene expression data were presented as mean ± standard deviation and a non-parametric Kruskal–Wallis analysis was performed followed by post-hoc pairwise comparisons (Mann–Whitney test). Histological semi-quantitative data were analysed by 2-way Fisher Exact Test to assess significant differences with respect to control group including samples assigned to a category without reference to severity gradations (total finding incidence). The Cochran-Armitage Trend Test was used to evaluate a dose–response trend. Differences among groups were considered significant if the *P*-value was < 0.05.

## Results

### General toxicity, body weight and food consumption

**Fig. 1:**
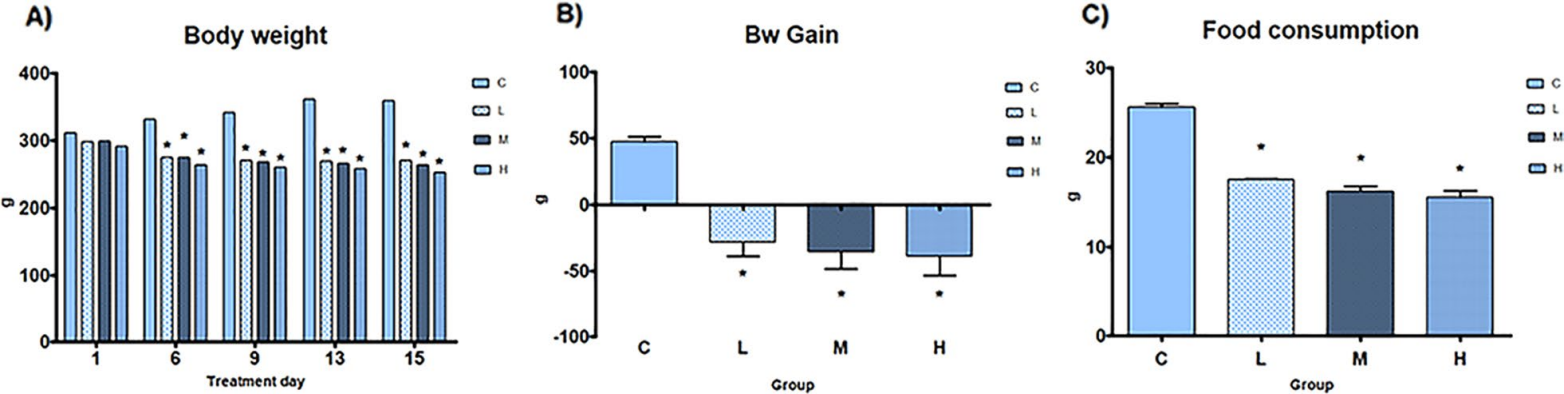
General toxicity data. General toxicity data of male rats subcutaneously treated with estradiol valerate plus cyproterone acetate, five times a week for 2 weeks: C: 0—sesame oil, Low (L) 0.045 + 0.2, Medium (M) 0.09 + 0.2 and High (H) 0.18 + 0.2 mg. Panel **A** Body weight (bw) at treatment days 6, 9, 13, 15. Panel **B** bw gain. Panel **C** food consumption. Data are presented as mean ± standard deviation. Statistical significance: * *p* < 0.05 Mann–Whitney test

No death or adverse clinical effects have been recorded. The bw at treatment days 6, 9, 13, 15, bw gain and food consumption were significantly decreased in all treatment groups in comparison to C (Fig. 1).

### Reproductive endpoints

**Fig. 2:**
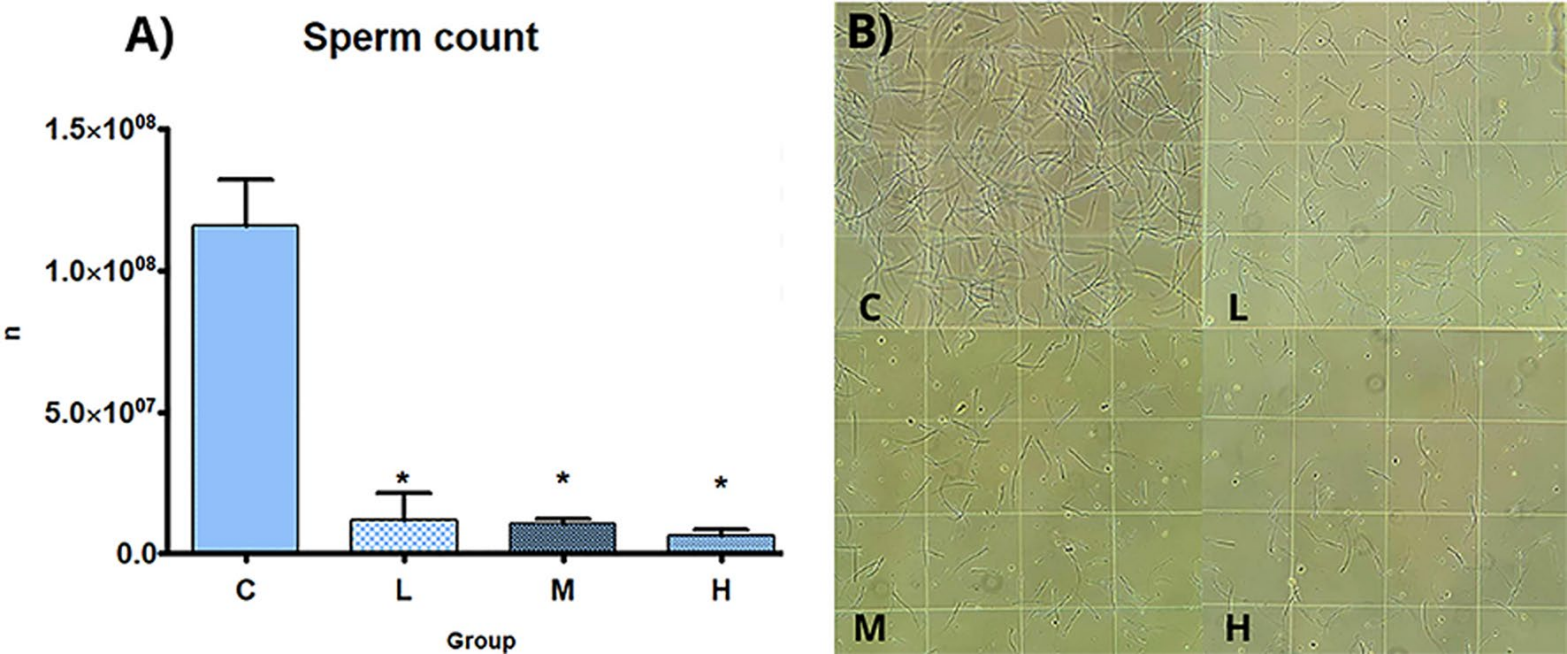
Sperm count. Sperm count of male rats subcutaneously treated with estradiol valerate plus cyproterone acetate, five times a week for 2 weeks C: 0—sesame oil, Low (L) 0.045 + 0.2, Medium (M) 0.09 + 0.2 and High (H) 0.18 + 0.2 mg. Panel **A** sperm count. Data are presented as mean ± standard deviation. Statistical significance: * *p* < 0.05 Mann–Whitney test. Panel **B** Light microscopic photos of sperm count on Neubauer chamber (original magnification 10 × ; area of 1/16 mm^2^)

**Fig. 3:**
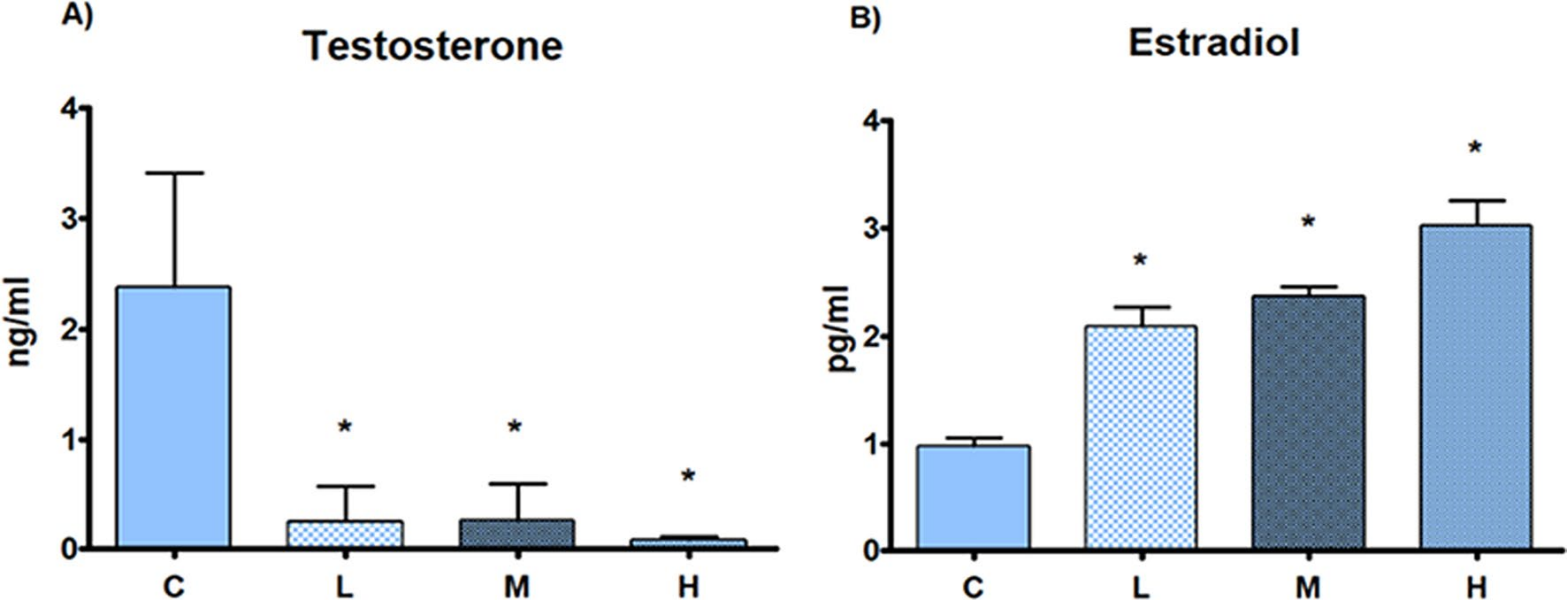
Reproductive hormone serum levels. Reproductive hormone serum levels of male rats subcutaneously treated with estradiol valerate plus cyproterone acetate, five times a week for 2 weeks: Control (C): 0—sesame oil, Low (L) 0.045 + 0.2, Medium (M) 0.09 + 0.2 and High (H) 0.18 + 0.2 mg. Panel **A** Testosterone. Panel **B** Estradiol. Data are presented as mean ± standard deviation. Statistical significance: * *p* < 0.05 Mann–Whitney test

Testis absolute weight was significantly decreased in all treatment groups in comparison to C; no differences in relative weight were seen among  treatment and C groups (Table 2). Sperm count was dose-dependently decreased, statistically significant in all treatment groups (Fig. 2). T serum levels were significantly decreased and E2 serum levels were statistically increased in all treatment groups in comparison to C group (Fig. 3).

**Table 2:** Absolute and relative weight of testes and liver

	C	L	M	H
N	4	4	4	4
Testis absolute weight (g; mean ± SD)	3.68 ± 0.11	2.55 ± 0.28*	2.55 ± 0.18*	2.37 ± 0.41*
Testis relative weight x 100 (mean ± SD)	1.03 ± 0.02	0.95 ± 0.11	0.96 ± 0.07	0.94 ± 0.15
Liver absolute weight (g; mean ± SD)	12.96 ± 0.84	10.62 ± 1.01*	10.38 ± 0.9*	10.65 ± 0.82*
Liver relative weight x 100 (mean ± SD)	3.61 ± 0.21	3.93 ± 0.31	3.94 ± 0.36	4.22 ± 0.26

Absolute and relative weight of testes and liver of male rats subcutaneously treated with estradiol valerate plus cyproterone acetate, five times a week for 2 weeks: Control (C): 0—sesame oil, Low (L) 0.045 + 0.2, Medium (M) 0.09 + 0.2 and High (H) 0.18 + 0.2 mg. Statistical significance: * *p* < 0.05 Mann–Whitney test. N: samples number; SD: standard deviation

**Fig. 4:**
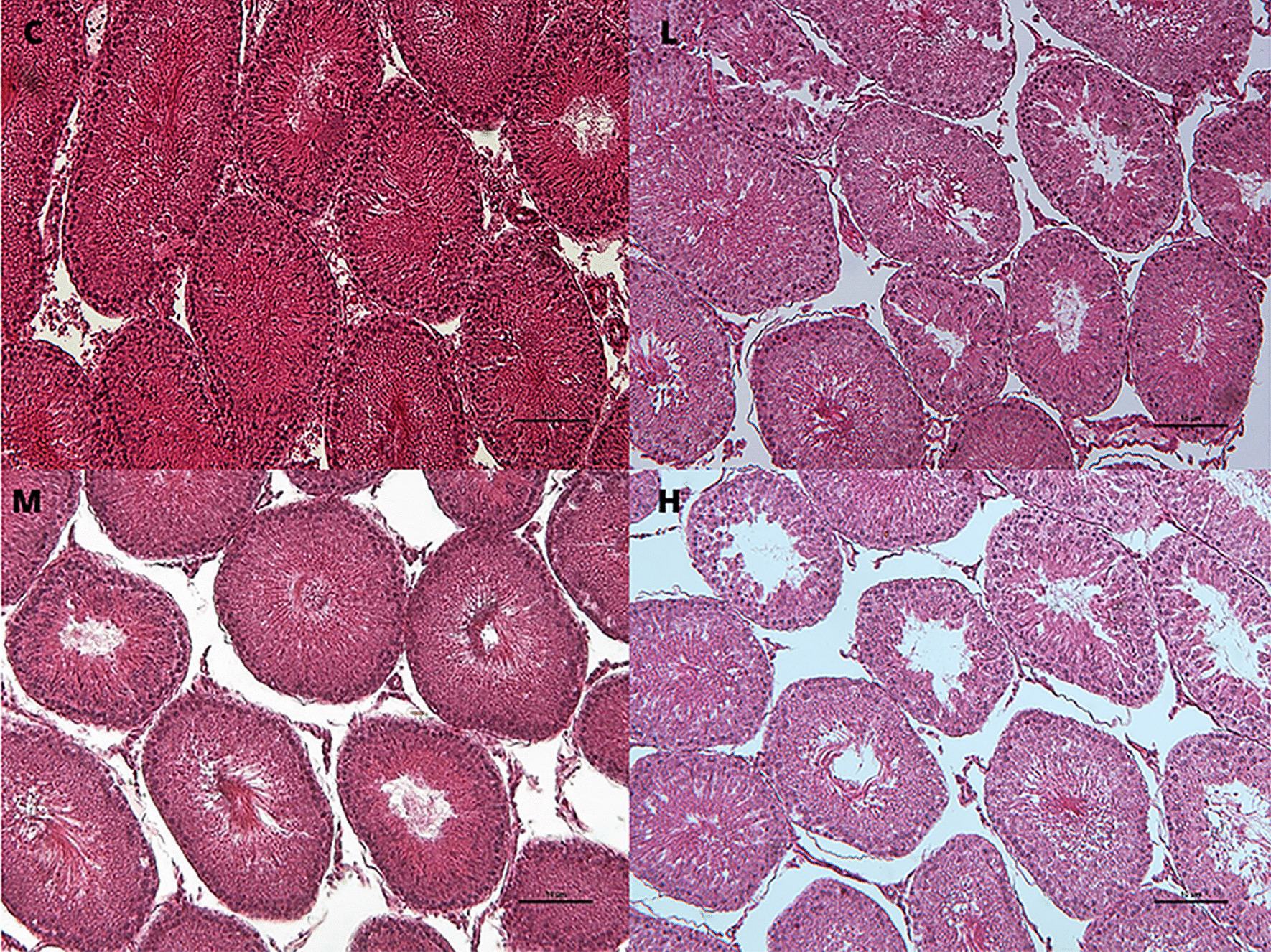
Testis histophatological features. Testis tubule degeneration with depletion of germ cells in male rats subcutaneously treated with estradiol valerate plus cyproterone acetate, five times a week for 2 weeks: Control (C): 0—sesame oil, Low (L) 0.045 + 0.2, Medium (M) 0.09 + 0.2 and High (H) 0.18 + 0.2 mg. Bar 10 μm (original magnification 10 × ; haematoxylin and eosin stain)

Histopathological analysis showed dose-dependent, significant increase of tubule degeneration in testes with depletion of germ cells (L, 1/4 sample: grade 1; M, 2/4 samples; grade 1; H, 2/4 samples:grade 1 plus 2/4 samples: grade 2) and statistically significant reduction of tubule lumen area in the H group compared to the C group (Table 3 and Fig. 4).

**Table 3:** Histopathological data

Organ/Observation		C	L	M	H
*Testis*	N	4	4	4	4
Tubule degeneration with germinal epithelium degeneration		0/4 ##	1/4 (25%)	2/4 (50%)	4/4 (100%) §
Tubule lumen area (μm^2^; mean ± SD)		133.00 ± 24.06	131.43 ± 30.4	172.35 ± 19.03	84.03 ± 9.39*
*Liver*	N	4	4	4	4
Sinusoidal dilatation		0/4 ##	2/4 (50%)	4/4 (100%) §	4/4 (100%) §
Hepatocyte vacuolization		0/4	1/4 (25%)	4/4 (100%) §	2/4 (50%)
*Thyroid*	N	4	3	4	3
Follicular number (mean ± SD)		28.67 ± 10.97	104.00 ± 3.61°	104.67 ± 34.53*	70.33 ± 44.05
Follicular density % (N/μm^2^; mean ± SD)		0.57 ± 0.21	1.93 ± 0.06°	1.90 ± 0.55*	1.73 ± 0.70°
Follicular area (μm^2^; mean ± SD)		49.63 ± 3.48	21.96 ± 9.70°	25.82 ± 5.76°	31.87 ± 9.91°
Colloidal area (μm^2^; mean ± SD)		32.49 ± 1.39	10.5 ± 7.75°	12.40 ± 6.37°	18.62 ± 7.78°
Direct follicular cell height (μm; mean ± SD)		8.92 ± 0.81	7.18 ± 1.17	9.20 ± 1.70	8.41 ± 0.89
Area of follicular epithelium (μm^2^; mean ± SD)		1.53 ± 0.14	2.40 ± 0.73	2.03 ± 0.77	1.76 ± 0.19

Histopathological data of testes, liver and thyroid of male rats subcutaneously treated with estradiol valerate plus cyproterone acetate, five times a week for 2 weeks: Control (C): 0—sesame oil, Low (L) 0.045 + 0.2, Medium (M) 0.09 + 0.2 and High (H) 0.18 + 0.2 mg. Statistical significance: § *p* < 0.05 Fisher exact test; ° *p* = 0.08, * *p* < 0.05 Mann–Whitney test; ## *p* < 0.01 Cochran–Armitage Trend Test. N: samples number; SD: standard deviation.

### Liver endpoints

Liver absolute weight was significantly decreased in all treatment groups in comparison to C, relative weight was unchanged (Table 2).

**Fig. 5:**
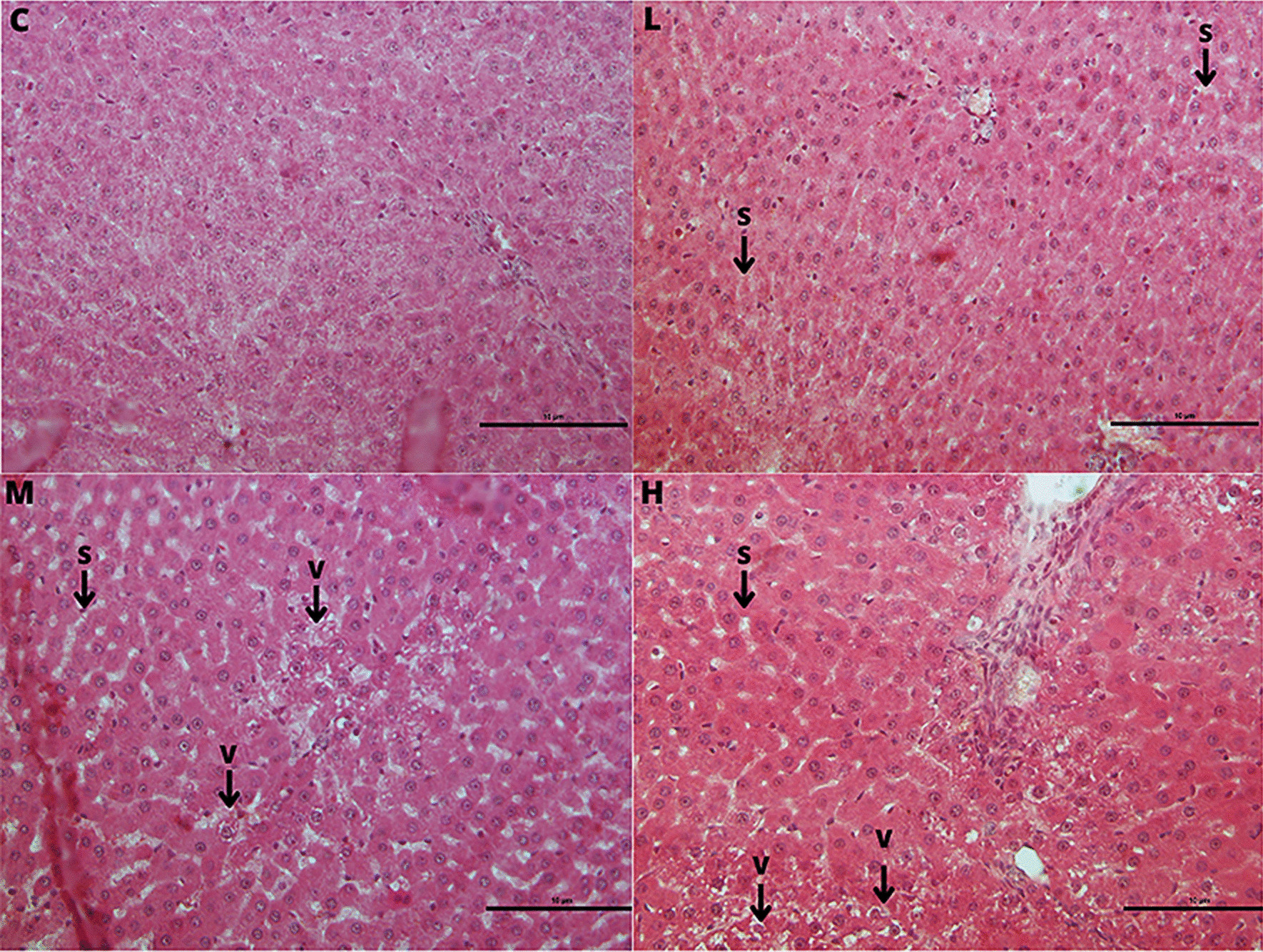
Liver histophatological features. Liver sinusoidal dilatation (S) and hepatocyte vacuolization (V) in male rats subcutaneously treated with estradiol valerate plus cyproterone acetate, five times a week, for 2 weeks: Control (C): 0—sesame oil, Low (L) 0.045 + 0.2, Medium (M) 0.09 + 0.2 and High (H) 0.18 + 0.2 mg. Bar 10 μm (original magnification 20 × ; haematoxylin and eosin stain)

Histopathological evaluation showed statistically significant increase of sinusoidal dilatation in M (1/4 samples: grade 1 plus 3/4 samples: grade 3) and H (3/4 samples: grade 1 plus 1/4 samples: grade 3) groups and of hepatocyte vacuolization in M (2/4 samples: grade 1 plus 2/4 samples: grade 2) group compared to C group (Table 3; Fig. 5).

**Fig. 6:**
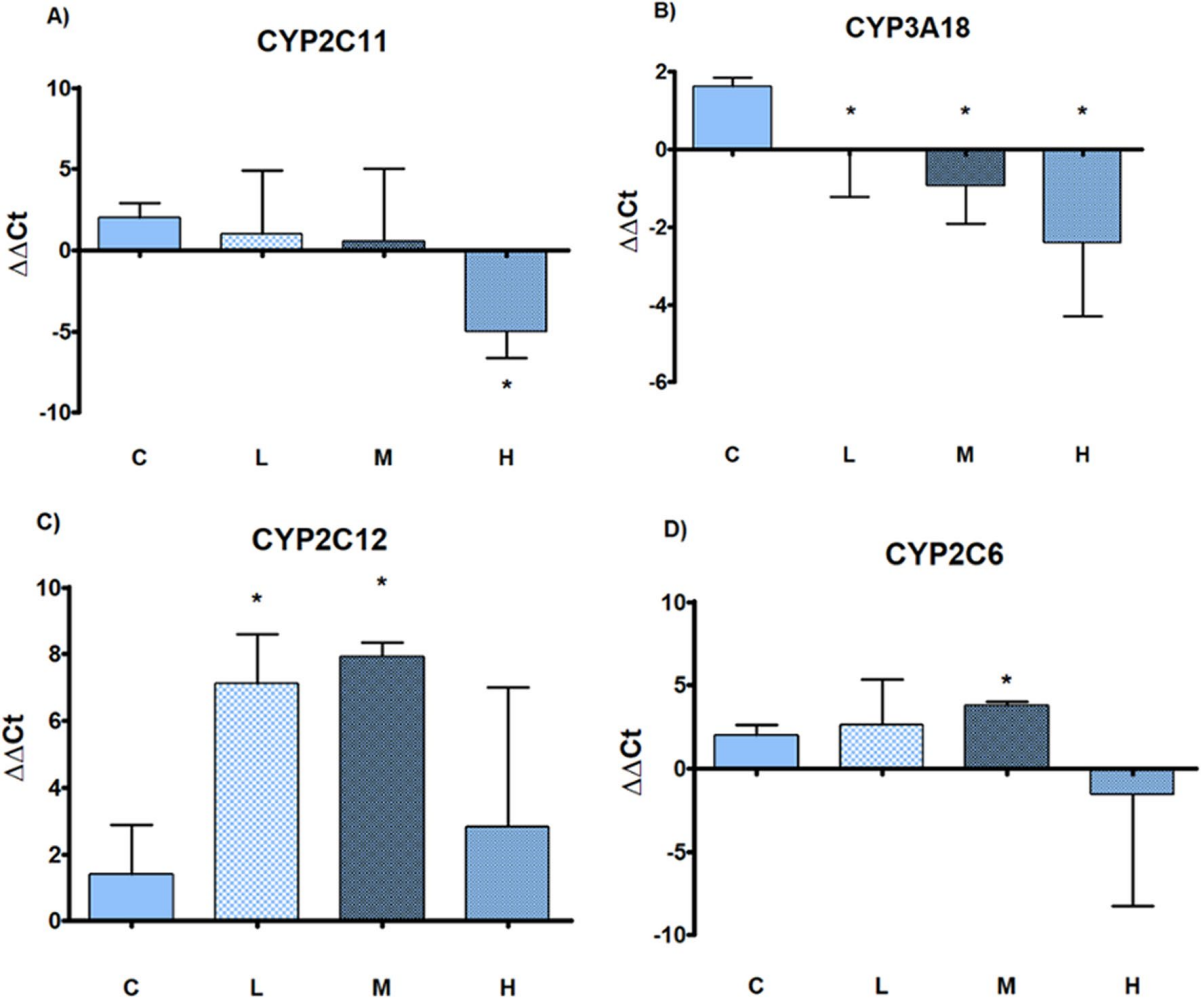
Gene expression. Gene expression analysis of sex specific CYP450 isoforms by real-time PCR in liver of male rats subcutaneously treated with estradiol valerate plus cyproterone acetate, five times a week for 2 weeks: Control (C): 0—sesame oil, Low (L) 0.045 + 0.2, Medium (M) 0.09 + 0.2 and High (H) 0.18 + 0.2 mg. Panel **A** *Cyp2c11*; Panel **B** *Cyp3a18*; Panel **C** *Cyp2c12*; Panel **D** *Cyp2c6*. Data are presented as mean ± standard deviation. Statistical significance: * *p* < 0.05 Mann–Whitney test

Gene expression analysis indicate that *Cyp2c11* (male specific isoform) was down-regulated in H group and *Cyp3a18* (male predominant isoform) was significantly down-regulated in all treatment groups (Fig. 6; Panels A and B). *Cyp2c12* (female specific isoform) was up-regulated in all treatment groups, statistically significant in L and M (Fig. 6; Panel C), the *Cyp2c6* (female predominant isoform) was significantly up-regulated in the M group in comparison to C (Fig. 6, Panel D).

### Thyroid endpoint

**Fig. 7:**
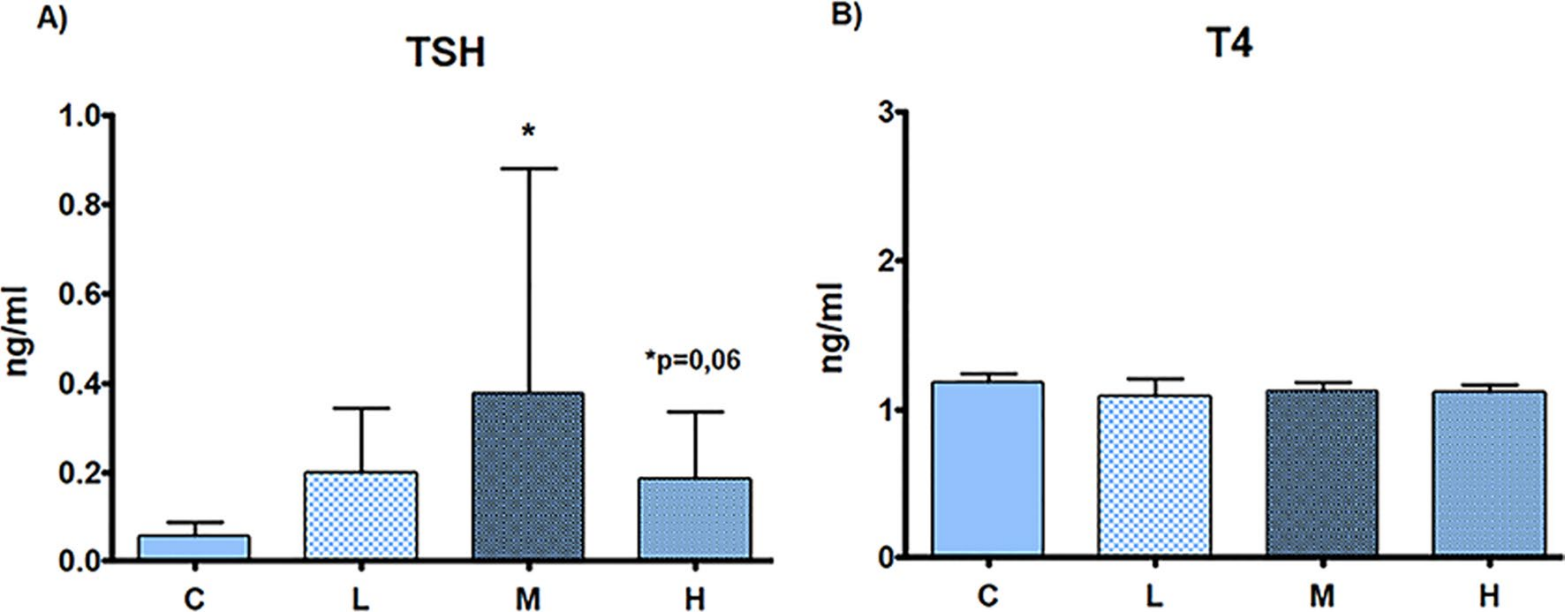
Thyroid biomarker serum levels. Serum levels of thyroid biomarkers of male rats subcutaneously treated with estradiol valerate plus cyproterone acetate, five times a week for 2 weeks: Control (C): 0—sesame oil, Low (L) 0.045 + 0.2, Medium (M) 0.09 + 0.2 and High (H) 0.18 + 0.2 mg. Panel **A** Thyroid-stimulating hormone (TSH). Panel **B** thyroxine (T4). Data are presented as mean ± standard deviation. Statistical significance: * *p* < 0.05 Mann–Whitney test

TSH serum levels was significantly increased in M and H (*p* = 0.06) treatment groups in comparison to C. No treatment-related alterations were observed in T4 serum levels (Fig. 7).

**Fig. 8:**
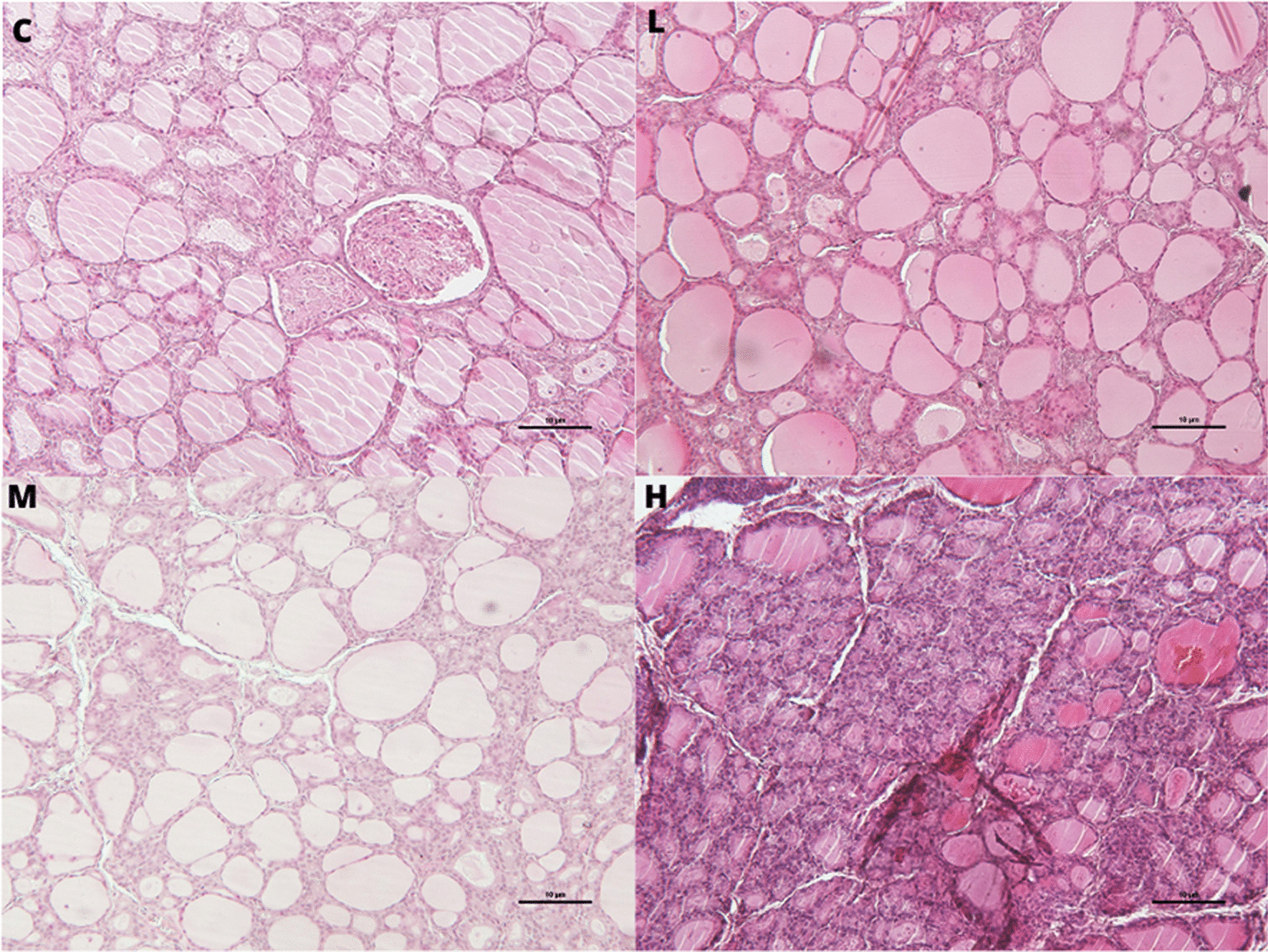
Thyroid histophatological features. Thyroid hypertrophy in male rats subcutaneously treated with estradiol valerate plus cyproterone acetate, five times a week for 2 weeks: Control (C): 0—sesame oil, Low (L) 0.045 + 0.2, Medium (M) 0.09 + 0.2 and High (H) 0.18 + 0.2 mg. Bar 10 μm (original magnification 10 × ; haematoxylin and eosin stain)

Histopathological analysis indicated follicular cell hypertrophy with central follicles tightly packed and smaller than normal in all treatment groups (Fig. 8). Follicular density was increased in all treatment groups with an increase of follicular number. The follicle dimension was decreased with reduction of both follicle and colloid areas in all treatment groups compared to the C. The epithelium cell height and area of follicles were unaffected (Table 3).

## Discussion

TG people often undertake a gender affirming path, which includes lifelong HT. Indeed, although HT has shown to have positive physical and psychological effects on the transitioning individual, scarce data are still available about its long-term outcomes. For this reason, it is crucial to implement an animal model that allows to explore in detail mechanisms and pathways linked to such aspects. Moreover, considering the potential different susceptibility and vulnerability of TG people to environmental contaminants, the animal model can represent a key tool for risk assessment purposes [6], and to obtain information supporting clinical studies e.g., concerning bone health [7] or filling data gap e.g., on thyroid homeostasis.

In a previous study aimed at setting the dMF animal model, although several endpoints were positively correlated with the switching towards demasculinization, the doses of E2 valerate plus CPA appeared to be too high, causing marked toxicity effects, and consequently the model could not be implemented and/or used in long term studies [10].

In the present experiment, as expected, after two weeks of feminizing HT, the male rats showed reduction of bw and food consumption in all treatment groups. The available in vivo studies described similar effects on bw in rats with up to 30 days of E2 plus CPA administration [10, 23]. Indeed, the lack of weight gain could reflect the estrogenic inhibition of eating [24] which can explain both the reduction of food intake and of bw observed in all treatment groups. Interestingly, in TG woman, a bw increase consisting in gain of body fat and decline in lean body mass was observed after the beginning of HT [25], while the body mass index becomes stable following 3 to 6 years [26]. Moreover, T levels decreased and E2 increased in all the treatment groups; in particular, the T levels of 0.08 to 0.26 ng/ml fell into the range of female serum levels [10]. In TG woman upon the initiation of E2 and CPA treatment, the serum concentration of E2 rises whereas T drops, approximating the female physiological sex hormone range [27].

Regarding the reproductive system, testis weight and sperm count were significantly reduced in all treatment groups, together with dose dependent histopathological pictures of tubule degeneration (C = 0%; L = 20%; M = 50%; H = 100%) which become significant at the highest dose. In the previous study, with higher daily doses of E2 valerate and CPA, the HT impaired the testicular architecture and the different testicular cellular types in all the samples at all dose levels [10]. Indeed, the effects recorded are consistent with the antiandrogenic activity of CPA (increased E2) [28] and of estrogen (tubular degeneration) [29]. The data of the present study, although substantially confirming the previous results, are less marked [10]; in addition, they indicated that the reproductive function was not completely altered in male rats after two weeks of HT. Concerning TG women, in literature scarce data are available about the effects of HT on testis morphology and spermatogenesis; the existing papers showed lacking and variable results, and the number of patients is very limited. An interesting study performed in 2019 on seventy-two adult TG women subjected to HT for > 1 year before orchiectomy, showed that the 80% of TG women had still germ cells and that spermatogenesis was preserved in approximately 40% of the patients; thus, the data suggested that duration of HT did not affect the degree of preservation of germ cells or the spermatogenesis, as could happen starting the hormonal treatment at a younger age. In humans, the volume of testes can be used as a marker to predict the presence of preserved spermatogenesis [30].

It has been already discussed that the liver plays a pivotal role in the sex hormone metabolism, e.g., producing carrier proteins that are important in reproduction and development [31]. In fact, in mammals, liver shows the highest degree of sexual dimorphism, with 72% of the genes expressed in a sexually differentiated manner, while in other organs the degree of sex dependent variability ranges between 14 and 60% [32]. In the previous study, the selected doses induced marked histopathological effects, in particular sinusoidal dilatation and hepatocyte vacuolation [10]. In this study, although similar signs of hepatotoxicity were still evident, they were milder and limited to M and H groups. On the other hand, despite the reduction of doses, the CYP gene expression showed once again to be valid biomarker for evaluating the success of HT. In fact, similarly to the previous data [10], signs of demasculinization in the expression of sex-specific CYPs were evident up to the lowest dose of E2 valerate (0.045 mg) and CPA (0.2 mg).

In addition, considering the complexity of endocrine system that controls body processes and functions and the well-known link between thyroid and gonads through the hypothalamic-pituitary gonadal axis [33], mainly in females but evident also in males [34, 35], it appeared of interest to investigate the potential impact of HT on thyroid homeostasis. At present, this is the first in vivo study that explores the potential impact of HT on thyroid. The results showed early signs of follicular cell hypertrophy—identified by quantitative histomorphometrical measurement on follicles—supported by increased TSH serum levels in M and H groups [36]. It is important to note that, in rodents, increased serum TSH levels and resultant follicular cell hypertrophy/hyperplasia are typical hormonal and histopathological findings attributable to compounds altering thyroid function [37]. Indeed, a higher number of animals might have highlighted significant alterations of TSH; nevertheless, the results obtained lead to hypothesize a decrease in thyroid activity. At present, no data are available to indicate the potential influence of gender affirming HT on thyroid homeostasis in TG woman and men. Recent research estimated an increased prevalence of almost 9% of thyroid disease in the TG population, however, due to an assessment bias, the data may be underestimated [18].

In this respect, the inclusion of thyroid homeostasis in the animal model of gender affirming HT can represent a valuable biomarker to explore and complete the data gaps on TG people health.

## Conclusions

Among the doses selected for this study, the lowest corresponding to the administration of 0.045 mg E2 valerate plus 0.2 mg CPA can be considered suitable for a long-term HT administration. In fact, after two weeks of administration, it: (i) allows the achievement of T serum levels in the range of the corresponding cisgender, and (ii) increases E2 serum levels and depresses the male reproductive function, without overt signs of toxicity; interestingly, these last are sex-transition biomarkers described also in TG women undergone gender-affirming HT [19]. Moreover, such dose induced liver demasculinization in the expression of sex-specific CYPs in the absence of tissue damage, proving the reliability of sex-dimorphic liver genes as biomarkers of sex transition in animal model [10].

Actually, no studies focused on the effect of HT on the thyroid of TG women are available, so it is essential to deepen further this aspect, in order to ensure accurate and personalized care for TG people.

## Abbreviations

TG: Transgender
HT: Hormone therapy
T: Testosterone
CPA: Cyproterone acetate
dMF: De-masculinizing feminizing
EDs: Endocrine disrupters
E2: Estradiol
CYP450: Cytochrome P450
C: Control group
L: Low dose
M: Medium dose
H: High dose
BW: Body weight
DMEM: Dulbecco's Modified Eagle Medium
TSH: Thyroid stimulating hormone
T4: Thyroxine
