# Correction: Risk assessment of transgender people: implementation of a demasculinizing–feminizing rodent model including the evaluation of thyroid homeostasis

**DOI:** 10.1186/s13062-024-00457-2

**Published:** 2024-01-30

**Authors:** Alessia Tammaro, Gabriele Lori, Andrea Martinelli, Luigia Cancemi, Roberta Tassinari, Francesca Maranghi

**Affiliations:** 1 Center for Gender-Specific Medicine, Istituto Superiore di Sanità, Rome, Italy; 2 Department of Biomedicine and Prevention, University of Rome “Tor Vergata”, Rome, Italy; 3 Experimental Animal Welfare Sector, Istituto Superiore di Sanità, Rome, Italy

**Correction: Biology Direct (2024) 19:5** 10.1186/s13062-023-00450-1

After publication of this article [1], it was brought to our attention that the first name and family name of all authors need to be exchanged, the correct author names should be:

Alessia Tammaro, Gabriele Lori, Andrea Martinelli, Luigia Cancemi, Roberta Tassinari and Francesca Maranghi

The original publication has been corrected.
